# Nomogram to predict prognosis in patients with posterior circulation acute ischemic stroke after mechanical thrombectomy

**DOI:** 10.3389/fneur.2024.1406882

**Published:** 2024-06-05

**Authors:** Jiayang Li, Jin Zhang, Changxin Li, Jun Li, Xupeng Wu, Shaoshuai Wang

**Affiliations:** Department of Neurology, First Hospital of Shanxi Medical University, Taiyuan, China

**Keywords:** posterior circulation acute ischemic stroke, mechanical thrombectomy, prognosis, symptomatic intracerebral hemorrhage, nomogram

## Abstract

**Purpose:**

This study aimed to investigate the risk factors of prognosis and hemorrhagic transformation after mechanical thrombectomy (MT) in patients with posterior circulation acute ischemic stroke (PC-AIS) caused by large vessel occlusion. We sought to develop a nomogram for predicting the risk of poor prognosis and symptomatic intracerebral hemorrhage (sICH) in patients with PC-AIS.

**Methods:**

A retrospective analysis was conducted on 81 patients with PC-AIS who underwent MT treatment. We collected clinical information from the patients to assessed sICH and prognosis based on CT results and National Institutes of Health Stroke Scale (NIHSS) scores. Subsequently, they were followed up for 3 months, and their prognosis was assessed using the Modified Rankin Scale. We used the least absolute shrinkage and selection operator (LASSO) and multivariate logistic regression to determine the factors affecting prognosis to construct a nomogram. The nomogram’s performance was assessed through receiver operating characteristic curves, calibration curves, decision curve analysis, and clinical impact curves.

**Results:**

Among the 81 patients with PC-AIS, 33 had a good prognosis, 48 had a poor prognosis, 19 presented with sICH, and 62 did not present with sICH. The results of the LASSO regression indicated that variables, including HPT, baseline NIHSS score, peak SBP, SBP CV, SBP SD, peak SBP, DBP CV, HbA1c, and BG SD, were predictors of patient prognosis. Variables such as AF, peak SBP, and peak DBP predicted the risk of sICH. Multivariate logistic regression revealed that baseline NIHSS score (OR = 1.115, 95% CI 1.002–1.184), peak SBP (OR = 1.060, 95% CI 1.012–1.111), SBP CV (OR = 1.296, 95% CI 1.036–1.621) and HbA1c (OR = 3.139, 95% CI 1.491–6.609) were independent risk factors for prognosis. AF (OR = 6.823, 95% CI 1.606–28.993), peak SBP (OR = 1.058, 95% CI 1.013–1.105), and peak DBP (OR = 1.160, 95% CI 1.036–1.298) were associated with the risk of sICH. In the following step, nomograms were developed, demonstrating good discrimination, calibration, and clinical applicability.

**Conclusion:**

We constructed nomograms to predict poor prognosis and risk of sICH in patients with PC-AIS undergoing MT. The model exhibited good discrimination, calibration, and clinical applicability.

## 1 Introduction

Acute ischemic stroke (AIS) is the leading cause of disability and mortality, posing a severe threat to human well-being (1). Research on the Chinese population reveals that, in 2020, there were 15.5 million cases of ischemic stroke among adults >40 years, with a prevalence rate of approximately 2.3/100 people (2). Over 30% of AIS cases result from large vessel occlusion (LVO) (3). LVO causes ischemia and hypoperfusion in the affected area, and timely restoration of perfusion to the ischemic area can maintain brain cell activity. Thrombolysis treatment by intravenous recombinant tissue plasminogen activator is accessible within a 4.5 h window from the onset of symptoms. However, it is not always feasible owing to missed time windows or contraindications. In recent years, mechanical thrombectomy (MT) development has provided new treatment options for patients with AIS. MT treatment is safe and effective for patients with anterior circulation AIS (AC-AIS), extending the treatment time to 6–24 h (4).

Compared with AC-AIS, posterior circulation AIS (PC-AIS) occurs in the vertebrobasilar system-supplied area, resulting in a worse prognosis (5). The early symptoms of PC-AIS are mild and unspecific, making identification challenging. Therefore, patients with PC-AIS have a lower rate of benefit from intravenous thrombolysis than those with anterior circulation AIS, with only 1/6 of patients benefiting from this treatment (6). It indicates that MT is an important treatment for patients with PC-AIS. However, the outcome of MT treatment in PC-AIS remains controversial, with significant differences observed in four randomized controlled trials (RCTs). In the BEST and BASIC studies, no significant difference was observed in prognostic outcomes between the MT and drug therapy alone groups.

In contrast, the BAOCHE and ATTENTION studies indicated a more favorable prognosis for patients who underwent MT treatment (7–10). Most patients with PC-AIS can achieve revascularization after MT treatment; however, only 38% experience a good prognosis (11). Therefore, exploring the factors influencing prognosis can help clinicians identify patients at high risk of poor prognosis and provide more timely interventions. There are fewer studies on the factors influencing the prognosis of PC-AIS patients undergoing MT treatment. A meta-analysis revealed that hypertension and diabetes were associated with poor prognosis (12). Gao et al.’s study identified baseline National Institutes of Health Stroke Scale (NIHSS) score, posterior circulation acute stroke prognosis early computed tomography (PC-ASPECT) score, and modified thrombolysis in cerebral infarction (mTICI) classification as independent risk factors affecting patients’ prognosis (13).

Notably, no study investigates the impact of post-operative medical management on the risk of patients’ prognosis, and predictive models for poor prognosis and sICH are yet to be constructed. This study addresses these gaps by investigating factors affecting the prognosis and sICH of patients with PC-AIS undergoing MT and constructs a nomogram applicable to clinical practice. We aimed to guide targeted clinical interventions to improve the management of patients with PC-AIS and enhance their post-treatment prognosis.

## 2 Materials and methods

### 2.1 Participants

We retrospectively gathered data from 81 patients diagnosed with PC-AIS admitted to the First Hospital of Shanxi Medical University between June 2020 and November 2023. The inclusion criteria were: (1) AIS patients with intracranial posterior circulation large vessel occlusion diagnosed by cranial computed tomography (CT) or magnetic resonance imaging (MRI), (2) those who underwent MT treatment, (3) age ≥18 years, and (4) signed informed consent from the patient or their family. Exclusion criteria comprised: (1) intracranial hemorrhage confirmed by cranial CT or MRI, (2) wake-up stroke or unclear onset time, and (3) discharge or death within 24 h post-surgery. This study was approved by the Ethics Committee of the First Hospital of Shanxi Medical University, and verbal informed consent was obtained from all participants via telephone.

### 2.2 Data collection

Data collection included baseline data, surgical data, post-operative clinical data, and the neurological recovery of patients assessed after 90 days. Baseline data included sex, age, onset time, blood pressure (BP) and blood glucose (BG) at admission, NIHSS score, PC-ASPECTS score, thrombolytic therapy, hypertension, diabetes, coronary artery disease, cerebrovascular disease, smoking, and drinking history. Surgical data included onset to puncture time, hospital arrival to puncture time (HPT), arterial puncture to reperfusion time, location of vascular occlusion, times of embolectomy, and mTICI classification. Data on whether endovascular treatment was performed after mechanical thrombectomy to address potential intracranial atherosclerotic stenosis (ICAS) were also collected.

Post-operative clinical data included: (1) Blood pressure (BP): readings were recorded during the first 24 h post-surgery. All patients were monitored, and a non-invasive BP monitor was used to measure their blood pressure every 4 h. To assess BP, we recorded the peak, minimum, and mean values of systolic blood pressure (SBP) and diastolic blood pressure (DBP). Mean arterial pressure (MAP) was calculated from SBP and DPB. Additionally, we analyzed the standard deviation (SD) and coefficient of variation (CV) of SBP and DBP. The CV is derived by dividing the SD of the BP measurements by their mean value; (2) Blood glucose (BG): During the first 24 h post-surgery, fingertip glucose measurement was used to measure all patients BG levels every 4 h. Glycated hemoglobin (HbA1c) levels were evaluated on the first day after admission. BG levels were assessed by calculating the mean, SD, CV, and stress hyperglycemia ratio (SHR). SHR is calculated by dividing the patient’s BG level at admission by their baseline BG level. The baseline BG can be estimated using the HbA1c level, with the baseline glucose estimated by the formula: (1.59 × HbA1c)–2.59; (3) Symptomatic intracranial hemorrhage (sICH): defined as a hemorrhage observed in the follow-up CT/MRI scan associated with a 4-point increase in the NIHSS score; (4) Good functional outcome: a professionally trained neurologist assessed and calculated modified Rankin Scale (mRS) scores for patients 90 days after enrollment via telephone or outpatient follow-up. The 90 day mRS scores ranging from 0–2 were defined as a good prognosis, while the scores between 3–6 were defined as poor prognoses. Patients in the good prognosis group were functionally independent and could look after themselves independently without assistance, whereas patients in the poor prognosis group had functional limitations.

### 2.3 Statistical analysis

Statistical analysis was carried out using SPSS and R software. The Kolmogorov–Smirnov test was used to analyze whether the data were normally distributed. Predictors of poor prognosis and symptomatic intracranial hemorrhage (sICH) in patients with posterior circulation acute ischemic stroke (PC-AIS) were determined through least absolute shrinkage and selection operator (LASSO) regression analysis. Afterward, multivariate logistic regression analysis was conducted using the results obtained. We employed a backward-conditional stepwise method to determine the best mode. A nomogram was constructed based on the predictors of poor prognosis identified in the final model, and internal validation was performed using the bootstrap method with 1,000 repeated samples. Receiver operating characteristic (ROC) curves were used to evaluate discrimination, while calibration curves were used to test the consistency between predicted and observed probability. Additionally, decision curve analysis (DCA) and clinical impact curve (CIC) were employed to evaluate the clinical applicability.

## 3 Results

### 3.1 General clinical characteristics

**Figure 1:**
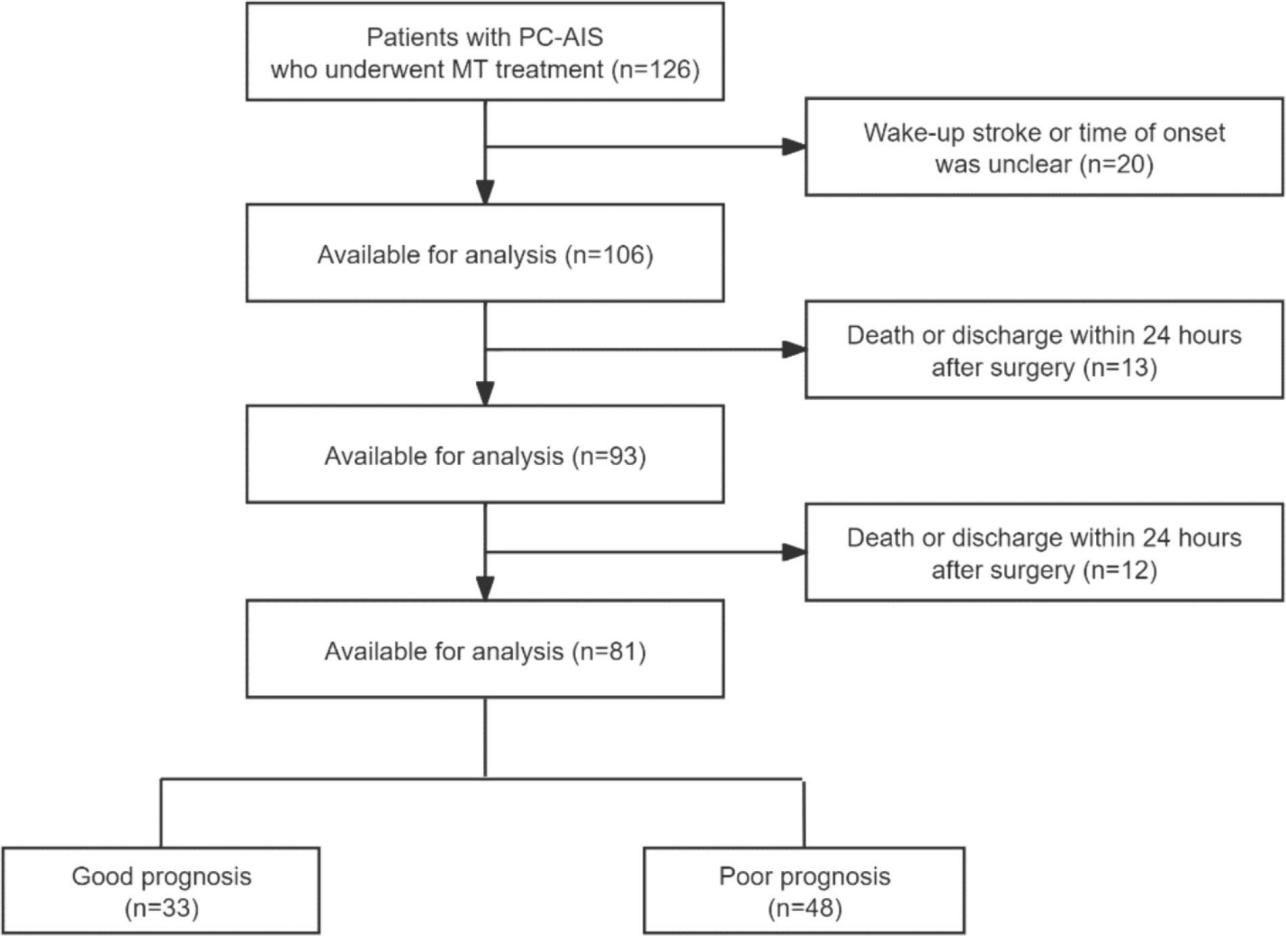
The flow chart for the inclusion of patients.

The flow chart is shown in Figure 1. This study included 81 patients with PC-AIS undergoing MT. Among them, 33 patients exhibited a good prognosis, while 48 had a poor prognosis. Table 1 presents the general clinical characteristics of all patients. The good prognosis group comprised 24 men and 9 women, while the poor prognosis group comprised 35 men and 13 women. Treatment outcomes indicated that 12 patients in the good prognosis group underwent successful revascularization, while 5 patients experienced sICH after the procedure. In contrast, in the poor prognosis group, 17 patients underwent revascularization surgery, and 14 experienced sICH. After regrouping the patients based on whether they experienced sICH, 19 patients had sICH, and 62 patients did not have sICH.

**Table 1:** Characteristics of all patients in prognosis group and ICH group.

	Prognosis		ICH	
	Good Prognosis (*n* = 33)	Poor prognosis (*n* = 48)	ICH (*n* = 19)	No ICH (*n* = 62)
Baseline characteristics				
Sex (Male)	24	35	12	15
Age	62.88 ± 11.77	68.96 ± 11.63	62 (52,68)	62 (55,70)
Hypertension	21	28	12	37
Diabetes	7	12	4	15
Coronary heart disease	6	10	2	14
Atrial fibrillation	9	15	11	13
Cerebrovascular disease	11	15	6	20
Smoking history	15	23	10	28
Drinking history	8	13	3	18
Time of onset	10.80 ± 6.07	9.74 ± 5.79	10 (6,15)	9.3 (5,16)
NHISS score	14.42 ± 8.00	22.74 ± 8.62	22.07 ± 8.28	18.52 ± 9.48
PC-ASPECTS	9 (8,10)	9 (8,10)	9 (7,10)	9 (8,10)
Thrombolysis	9	7	3	13
admission SBP	145.24 ± 27.50	146.60 ± 24.13	146.16 ± 23.35	146.60 ± 24.13
admission DBP	88 (81.5,98)	88 (73,91.5)	88 (81.5,98)	88 (73,91.5)
admission BG	6.9 (6,8.6)	7.1 (6.2.9.15)	6.5 (5.9,8)	7.1 (6.35.9.38)
Lesion location				
Brainstem	19	27	16	30
Cerebellum	22	32	16	38
Thalamus	6	13	4	15
PCA supply area	15	25	10	30
Occlusion sites				
Basilar artery	24	34	14	44
Vertebral artery	9	14	5	18
TOAST classification				
Large artery atherosclerosis	17	18	7	28
Cardioembolism	11	23	10	24
Others	5	7	2	10
Surgical data				
OPT	451 (330,762)	540 (416,1018.75)	411 (375,879)	540 (393.75,908.75)
HPT	125 (92,163)	161 (119,227)	132 (100,224)	147 (114,211.25)
PRT	88 (59,103)	82 (58.5,107)	81.58 ± 27.77	86.58 ± 29.84
Number of MT	1 (1,1)	1 (1,2)	2 (1,2)	1 (1,1)
mTICI				
0-2a	4	5	2	7
2b-3	29	43	18	54
Endovascular therapy	12	17	10	19
sICH	5	14	—	—
Postoperative data				
peak SBP	148.94 ± 16.00	164.20 ± 16.69	172.13 ± 19.16	153.65 ± 15.29
peak DBP	95.10 ± 8.45	95.27 ± 6.54	99.95 ± 5.85	93.75 ± 7.15
SBP mean	138 (128,146)	143 (132.5,148)	142 (131,156)	142 (130,146.25)
DBP mean	80 (77.5,85.5)	84 (78,85.5)	84 (77.5,86)	81.67 (77.83,85.34)
MAP	100 (95,104.33)	102 (98,105.75)	104.45 (96,107)	100.89 (97.41,104.39)
SBP SD	7.69 (4.90,10.18)	11.44 (7.54,16.4)	11.3 (7.69,16.6)	8.62 (7.02,12.79)
DBP SD	6.67 (4.37,9.09)	9.83 (6.18,12.99)	9.35 (4.47,14.7)	7.63 (5.67,11.05)
SBP CV	7.59 ± 3.30	9.67 ± 3.42	9.07 ± 3.08	8.75 ± 3.65
DBP CV	8.40 ± 3.88	11.74 ± 4.51	10.85 ± 5.72	10.24 ± 4.17
BG mean	7.56 (6.99,8.22)	7.81 (6.88,9.28)	7.85 (7.17,8.27)	7.69 (6.89,8.91)
BG SD	0.83 (0.59,1.09)	0.82 (0.63,1.17)	0.87 (0.71,1.12)	0.78 (0.61,1.16)
BG CV	11 (8.5,14)	10.50 (7.25,14)	11 (9,15)	11 (8,14)
HbA1c	5.47 (5.09,6.18)	6.55 (5.67,8.18)	6.54 (5.2,7.32)	5.90 (5.26,7.31)
SHR	0.67 (0.29,1.1)	0.72 (0.21,0.97)	0.8 (0.37,1.02)	0.66 (0.21,1.05)

SBP, systolic blood pressure; DBP, diastolic blood pressure; OPT, onset to puncture time; HPT, hospital arrival to puncture time; PRT, arterial puncture to reperfusion time; mTICI, modified thrombolysis in cerebral infarction; sICH, symptomatic intracranial hemorrhage; CV, coefficient of variation; SD, standard deviation; BG, blood glucose; SHR, stress hyperglycemia ratio; HbA1c, glycated hemoglobin.

### 3.2 Screening of predictors and construction of prediction model

**Figure 2:**
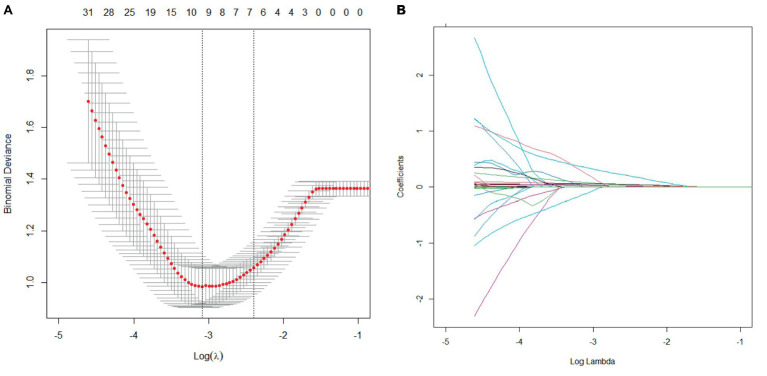
Cross-validation and lasso regression for predicting patient prognosis. **(A)** Cross-validation process; **(B)** Lasso regression analysis.

**Figure 3:**
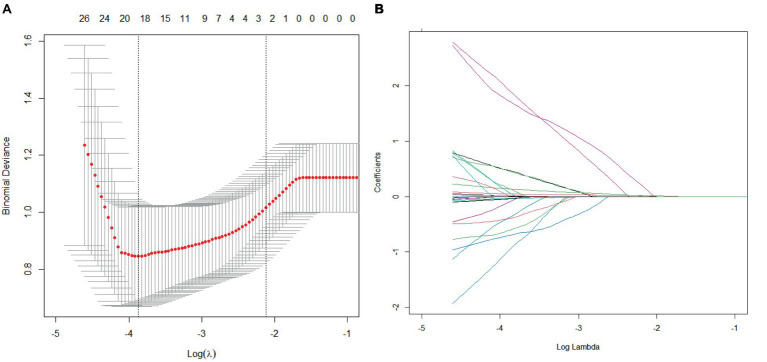
Cross-validation and lasso regression for predicting sICH. **(A)** Cross-validation process; **(B)** Lasso regression analysis.

The LASSO regression method was used to screen the predictors. We first determined the optimal value of *λ* through cross-validation, as illustrated in Figures 2A, 3A. In the process of selecting *λ*, we adhered to the 1se rule. According to this rule, setting *λ* to 0.09 identified seven variables for predicting patient prognosis, including HPT, baseline NIHSS score, peak SBP, SBP CV, SBP SD, DBP CV, HbA1c, and BG SD. When *λ* was set to 0.12, it identified three variables for predicting sICH, including atrial fibrillation, peak SBP, and peak DBP (Figures 2B, 3B).

Logistic regression models were then constructed using these variables. The results revealed that baseline NIHSS score, SBP CV, peak SBP, and HbA1c independently influenced prognosis factors. Specifically, higher pre-operative NIHSS scores (OR = 1.115, 95% CI 1.002–1.184), peak SBP (OR = 1.060, 95% CI 1.012–1.111), SBP CV (OR = 1.296, 95% CI 1.036–1.621), and HbA1c (OR = 3.139, 95% CI 1.491–6.609) were associated with worse outcomes (Table 2). Atrial fibrillation (OR = 6.823, 95% CI 1.606–28.993), peak SBP (OR = 1.058, 95% CI 1.013–1.105), and peak DBP (OR = 1.160, 95% CI 1.036–1.298) were associated with the risk of developing sICH (Table 3). The final model was constructed using these variables.

**Table 2:** Multifactor logistic regression results of poor prognosis in patients with PC-AIS.

	*β*	*p*	OR	95% CI	
				Lower	Upper
NHISS score	0.097	0.008	1.102	1.026	1.184
Peak SBP	0.058	0.015	1.060	1.012	1.111
SBP CV	0.259	0.023	1.296	1.036	1.621
HbA1c	1.144	0.03	3.139	1.491	6.609

SBP, systolic blood pressure; CV, coefficient of variation; HbA1c, glycated hemoglobin.

**Table 3:** Multifactor logistic regression results of ICH in patients with PC-AIS.

	*β*	*p*	OR	95% CI	
				Lower	Upper
Atrial fibrillation	1.920	0.009	6.823	1.606	28.993
Peak SBP	0.057	0.010	1.058	1.013	1.105
Peak DBP	0.148	0.010	1.160	1.036	1.298

SBP, systolic blood pressure; DBP, diastolic blood pressure.

### 3.3 Construction and validation of nomograms for predicting prognosis and sICH of patients with PC-AIS

**Figure 4:**
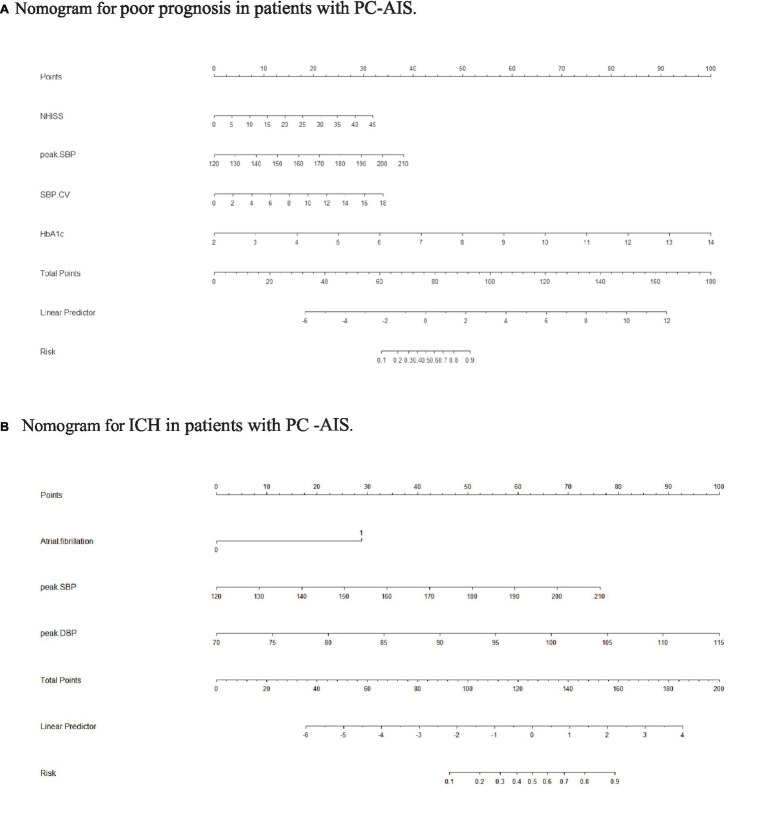
Nomogram. **(A)** Nomogram for poor prognosis in patients with PC-AIS. To estimate the risk of a PC-AIS patient having a poor prognosis after 3 months, find his/her value on each variable axis. Draw a vertical line from that value to the top of the point scale to determine the number of points assigned to that variable value. Then, add up the number of points for each variable value. Find the sum on the total points scale and project it vertically onto the lower axis to obtain the individualized risk of poor prognosis. SBP, systolic blood pressure; CV, coefficient of variation; BG, blood glucose; SD, standard deviation. **(B)** Nomogram for ICH in patients with PC-AIS. To estimate the risk of a PC-AIS patient having a poor prognosis after 3 months, find his/her value on each variable axis. Draw a vertical line from that value to the top of the point scale to determine the number of points assigned to that variable value. Then, add up the number of points for each variable value. Find the sum on the total points scale and project it vertically onto the lower axis to obtain the individualized risk of poor prognosis. SBP, systolic blood pressure; CV, coefficient of variation; BG, blood glucose; SD, standard deviation.

**Figure 5:**
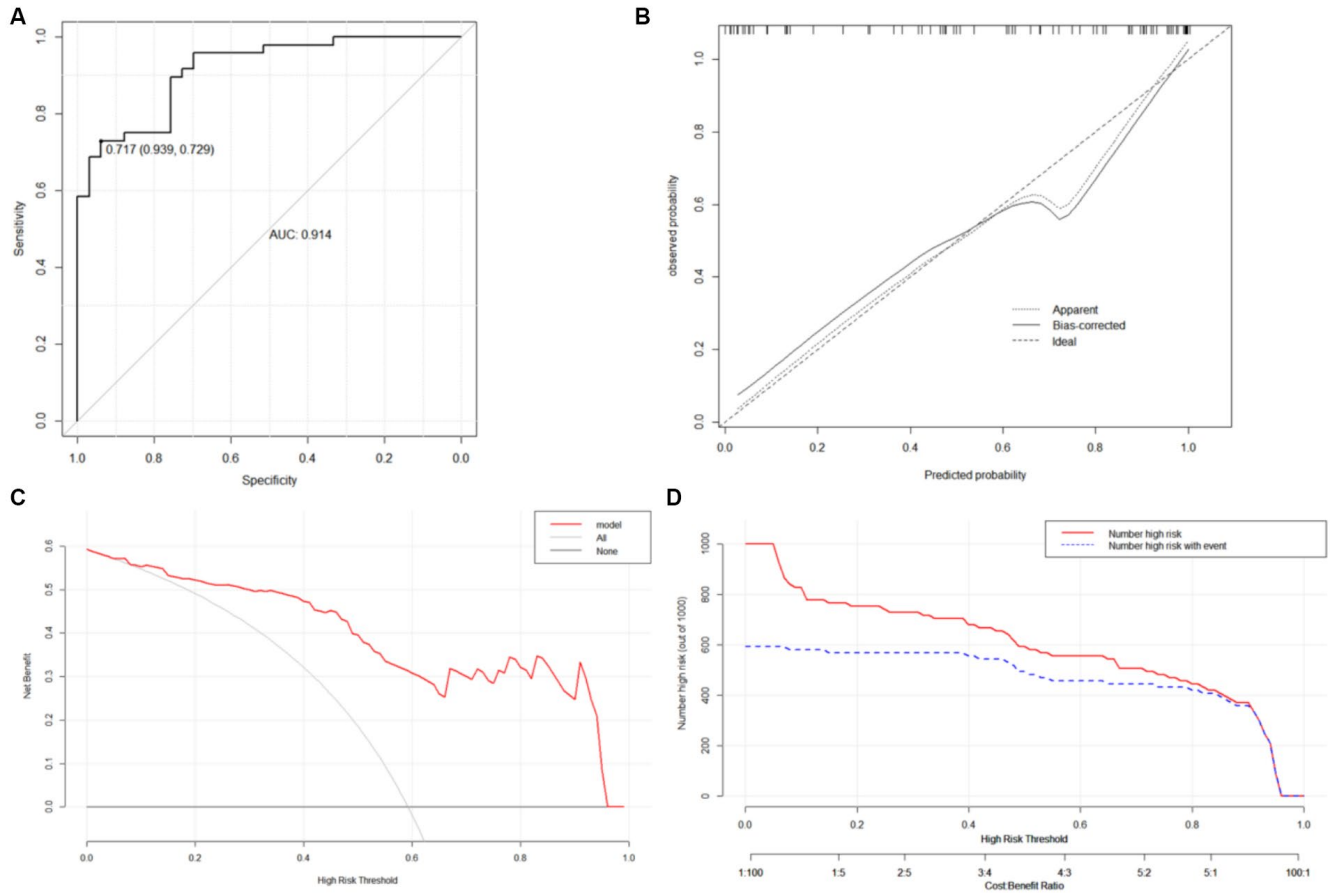
ROC curves **(A)**, calibration curves **(B)**, DCA curve **(C)** and CIC **(D)** of the nomogram A. **(C)** DCA curve: The horizontal axis is the threshold used to define high risk and the vertical axis is net benefit. **(D)** CIC: the red curve represents the number of people classified as positive (high risk) by the model at each threshold probability; the blue curve represents the number of true positives at each threshold probability.

**Figure 6:**
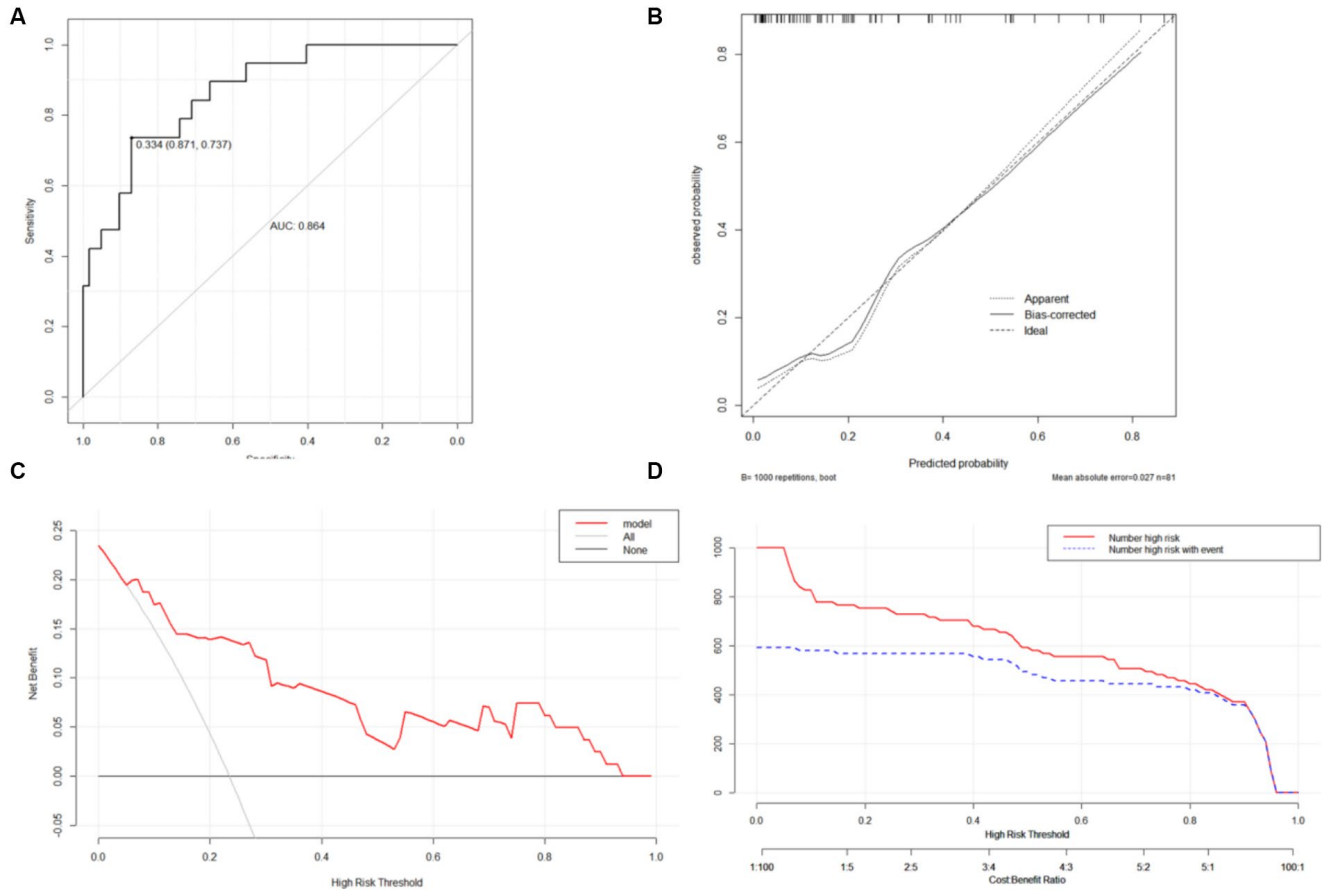
ROC curves **(A)**, calibration curves **(B)**, DCA curve **(C)** and CIC **(D)** of the nomogram B.

Nomograms for predicting prognosis and sICH were plotted in R software based on the independent influences identified in the results of multivariate logistic regression (Figure 4). The scores for the predictors ranged from 0–100. After summing the scores of all variables into a “total score,” the corresponding value represented the risk of poor prognosis and sICH. For example, if a patient has an NIHSS score of 29, a peak SBP of 160, an SBP CV of 8, and an HbA1c of 6, their total score is 20.58 + 16.96 + 15.10 + 33.33 = 85.97, indicating a corresponding risk of poor prognosis between 0.7 and 0.8. Similarly, if a patient has atrial fibrillation, a peak SBP of 180, and a peak DBP of 100, their total score is 20.58 + 28.81 + 50.87 + 66.67 = 146.35, indicating a corresponding risk of sICH between 0.2 and 0.3. Using the ROC curve, we validated the performance of the nomogram to evaluate the predictive model’s ability. The result demonstrated that the area under the curve (AUC) for prognosis was 0.914, and for the sICH, it was 0.864, confirming the good discriminatory ability of the nomogram (Figures 5A, 6A). Using the maximum Youden index, we set optimal cut-off values of the nomograms’ predicted probability *t* at 0.717 and 0.334.

The bootstrap method was used to validate the constructed models internally. Calibration curves for the models demonstrated excellent agreement between prediction and observation (Figures 5B, 6B). Additionally, DCA demonstrated a wide range of clinical applications for the models (Figures 5C, 6C). Based on DCA, the CIC was plotted to provide a more intuitive assessment of the clinical effect of the models (Figures 5D, 6D).

## 4 Discussion

Recently, MT has been used to treat AIS with increasing success. Despite its demonstrated superiority over drug treatment alone for patients with PC-AIS (14), the prognosis for most patients with PC-AIS remains poor, with a mortality rate of >30% and only 38% showing good prognosis (11). Our results showed that baseline NIHSS score, SBP CV, peak SBP, and HbA1c were identified as the independent risk factors for the prognosis of patients with PC-AIS. Atrial fibrillation, peak SBP, and peak DBP were independent risk factors for sICH. The nomograms were constructed to predict the prognosis and sICH of patients. These nomograms have good discrimination calibration and can be used as a valid tool in clinical practice.

The early recognition of PC-AIS is difficult, but the progression is rapid. One important indicator of stroke severity is the NIHSS score in patients. In patients with AC-AIS, the risk of poor prognosis after revascularization increases progressively as the NIHSS score rises (15, 16). However, few studies have investigated the effects of MT on patients with PC-AIS. Notably, the ENDOSTROKE study revealed that a low NIHSS score was an independent predictor of patient prognosis after 90 days of basilar artery occlusion (BAO) (17). Another study of 117 patients with BAO demonstrated that the baseline NIHSS score predicted mortality in patients who achieved successful revascularization (18). This study’s baseline NIHSS score was an independent risk factor for poor prognosis in patients with PC-AIS undergoing MT. As the NIHSS score increases by one point, the risk of poor prognosis increases by 0.102-fold. This finding highlights the importance of the NIHSS score as a prognostic assessment for patients with PC-AIS. Despite the greater risk of poor prognosis post-MT in patients with high NIHSS scores, there is still a chance of reperfusion of ischemic brain tissue and recovery of neurological function compared with drug therapy alone. A meta-analysis revealed that MT treatment was more beneficial than drug therapy alone for patients with NIHSS scores >20 (19). Patients with high NIHSS scores should not be excluded from MT treatment.

The focus of current research into MT treatment in patients with PC-AIS has primarily been on its safety and efficacy. However, post-operative patient management has received little attention. AIS is often associated with hypertension, and elevated BP variability is associated with greater mortality and disability following a stroke (20). Our study identified SBP CV as an independent risk factor for poor prognosis in PC-AIS with MT. In patients with AC-AIS, similar results were observed, where higher systolic blood pressure variability was a predictor of poor prognosis after MT and was associated with higher mortality (21, 22). This may be attributed to impaired cerebral autoregulation following AIS. Cerebral autoregulation maintains stable cerebral blood flow in the presence of fluctuations in systemic BP (23). This ability can protect the brain from the negative effects of blood pressure variations. When cerebral autoregulation is impaired, higher blood pressure variability can worsen ischemic and reperfusion injury, which can lead to complications such as hemorrhagic infarction and cerebral edema (24). These complications can ultimately lead to poor clinical outcomes. Therefore, maintaining a stable BP post-MT treatment is crucial in improving patient prognosis.

Elevated blood pressure increases the risk of reperfusion injury (25). Higher systemic arterial BP was associated with poor prognosis and a high risk of hemorrhage in patients with AC-AIS with MT treatment (26–28). In our study, peak SBP was an independent risk factor for poor prognosis and sICH, and peak DBP was an independent risk factor for sICH. The results of the ENCHANTED study revealed that intensive antihypertensive lowering to 140 mmHg did not enhance the prognosis of patients (29). This may be because rapid antihypertensive lowering increases the risk of hypoperfusion. Additionally, even with successful revascularization, some patients may still require higher BP levels to maintain normal perfusion. The current guidelines recommend maintaining SBP at 180 mmHg for 24 h post-MT (30), while the BEST study suggests that patients with AC-AIS with peak SBP exceeding 154.5 mmHg may have an increased risk of poor prognosis. However, there are no studies on post-operative BP management goals for patients with PC-AIS. In our study, we observed that the peak SBP of 161.5 mmHg was observed to best distinguish the prognostic outcome of patients. When the peak SBP exceeded this level, patients were at an increased risk of poor prognosis. Additionally, patients with a peak SBP >167.5 mmHg or peak DBP >92.5 mmHg have a higher likelihood of experiencing ICH within 24 h post-MT. Therefore, the management of SBP and DBP should be taken seriously in patients with AIS post-MT to reduce the risk of poor prognosis and sICH and to improve the quality of life of patients.

Additionally, our study results show that HbA1c is an independent risk factor for poor prognosis post-MT, and AF is an independent risk factor for sICH. HbA1c levels reflect the average blood sugar level over the past 2–3 months. Elevated HbA1c levels, indicating suboptimal long-term glucose management, emerge as a modifiable factor that warrants attention in the pre- and post-operative phases. Poor BG control can worsen ischemic injury through various mechanisms, such as disrupting the blood–brain barrier, increasing oxidative stress, exacerbating the thrombo-inflammatory cascade, and impairing fibrinolysis (31, 32). Ultimately, this leads to an expansion of infarct size and cerebral hemorrhage (33). Therefore, it is crucial to control blood glucose levels to improve patient prognosis.

Additionally, the independence of AF as a risk factor underscores the importance of vigilant monitoring and tailored interventions for individuals with this cardiac arrhythmia. Previous studies have identified that atrial fibrillation is an independent risk factor for hemorrhagic transformation in patients with AIS treated with intravenous thrombolysis or MT (34, 35), aligning with our findings.

However, the limitations to our study are as follows: (1) the small sample size of this study may influence the results; (2) as the patients in our study had high NIHSS scores, our findings may not apply to those with mild PC-AIS. Therefore, a larger multicenter study is required to confirm the results of the present study.

## 5 Conclusion

Our study aimed to identify factors influencing the prognosis of patients with posterior circulation AIS. Our results highlighted that the baseline NIHSS score, peak SBP, SBP CV, and HbA1c were independent factors affecting prognosis. AF, peak SBP, and peak DBP were associated with the risk of sICH. Furthermore, nomograms were developed to predict the prognosis and sICH for patients with PC-AIS. The models demonstrated good discrimination, calibration, and clinical applicability.

## Data availability statement

The raw data supporting the conclusions of this article will be made available by the authors, without undue reservation.

## Ethics statement

The studies involving humans were approved by the Ethics Committee of the First Hospital of Shanxi Medical University. The studies were conducted in accordance with the local legislation and institutional requirements. The participants provided their written informed consent to participate in this study.

## Author contributions

JiL: Data curation, Formal analysis, Methodology, Software, Validation, Visualization, Writing – original draft. JZ: Conceptualization, Funding acquisition, Methodology, Supervision, Writing – review & editing. CL: Formal analysis, Funding acquisition, Supervision, Validation, Writing – review & editing. JuL: Data curation, Methodology, Resources, Software, Writing – review & editing. XW: Data curation, Project administration, Resources, Validation, Writing – review & editing. SW: Conceptualization, Data curation, Funding acquisition, Investigation, Project administration, Resources, Supervision, Writing – review & editing.

## Glossary

AIS: acute ischemic stroke
LVO: large vessel occlusion
MT: mechanical thrombectomy
NIHSS: National Institutes of Health Stroke Scale
CT: computed tomography
MRI: magnetic resonance imaging
SBP: systolic blood pressure
DBP: diastolic blood pressure
PC-AIS: posterior circulation acute ischemic stroke
AC-AIS: anterior circulation acute ischemic stroke
mTICI: modified thrombolysis in cerebral infarction
BP: blood pressure
BG: blood glucose
ICAS: intracranial atherosclerotic stenosis
SD: standard deviation
CV: coefficient of variation
sICH: symptomatic intracranial hemorrhage
mRS: modified Rankin scale
ROC: receiver operating characteristic
DCA: decision curve analysis
CIC: clinical impact curve
HPT: hospital arrival to puncture time
BAO: Basilar artery occlusion
ENDOSTROKE: endovascular treatment for symptomatic vertebrobasilar occlusion
ENCHANTED: enhanced control of hypertension and thrombolysis stroke study
AUC: area under the curve
